# Exploring the Peer Effect of Physicians’ and Patients’ Participation Behavior: Evidence from Online Health Communities

**DOI:** 10.3390/ijerph19052780

**Published:** 2022-02-27

**Authors:** Qiuju Yin, Haoyue Fan, Yijie Wang, Chenxi Guo, Xingzhi Cui

**Affiliations:** 1 School of Management and Economics, Beijing Institute of Technology, Beijing 100089, China; yinqiuju@bit.edu.cn (Q.Y.); 3120215817@bit.edu.cn (H.F.); 2 Sustainable Development Research Institute for Economy and Society of Beijing, Beijing 100081, China; 3 School of Foreign Languages, Tianjin Normal University, Tianjin 300387, China; wangyijie@tjnu.edu.cn; 4 International Department, Beijing 101 Middle School, Beijing 100084, China; tigercui@yeah.net

**Keywords:** online health communities, peer effect, physicians’ knowledge sharing behavior, patients’ services evaluating behavior

## Abstract

Background: Little research has studied the peer effect of physicians and patients in online health communities (OHCs) simultaneously. The study investigates the impact of the focal physician’s peers (F-peers) on the focal physician (F-physician), and the impact of patients of the focal physician’s peers (F-P-patients) on the focal physician’s patients (F-patients). Moreover, based on brand extension and accessible–diagnosable theories, this study explores the moderating effects of the intensity of F-peers’ knowledge sharing behavior and department reputation. Methods: This study collects data of 3297 physicians and related patients from Haodf.com platform between January 2019 and December 2019. Both two-way fixed effect and panel negative binomial regression are adopted to quantify the effects. Results: Results show that the behavior of F-peers positively affects the behavior of the F-physician, while the behavior of F-P-patients positively affects the behavior of F-patients. Moreover, both the intensity of F-peers’ knowledge sharing behavior and department reputation have a compound moderating effect. Conclusions: This study contributes to the literature of peer effects by constructing the conceptual framework of different types of individual participation behaviors in OHCs. The findings offer practical guides for establishing an incentive mechanism and formulating peer incentives or competition strategies in OHCs.

## 1. Introduction

### 1.1. Background

Online health communities (OHCs) are online social networks in which patients and their families can consult online, make offline appointments and share their experiences with others. With the development of technology and the concern of health issues, OHCs are gradually emerging in many countries, such as *Doctor on Demand* in America, *Covidom* in France, *Haodf* in China, etc. The emergence of OHCs provides physicians and patients with a variety of different services, including appointments, health information queries, online consultations and referrals, etc. With the rapid development of OHCs, it has become an important tool for people to discuss health problems, share and search health knowledge, diagnose and treat diseases, make appointments and register [1,2]. It not only alleviates the problem of “difficult and expensive medical treatment” for patients, but also provides a new way of working for physicians without time and space constraints [3].

The development of OHCs depends on the participation of physicians and patients. Physicians can provide online consultation services to patients and share knowledge in OHCs, while patients can post reviews about physicians and experiences in OHCs, even rate the services quality of physicians based on their satisfaction. At the same time, physicians and patients are not independent individuals. However, some physicians no longer pay attention to the OHCs after registration, meaning little interaction with patients, little participation in knowledge sharing and a low amount of online consultations. A large number of patients leave the platform after receiving few consultations and do not actively publish services evaluations anymore. All these phenomena lead to the insufficient development of OHCs. Thus, it is necessary to explore the motivations of physicians’ and patients’ participation behavior in OHCs.

Normally, participation behavior in OHCs includes the knowledge-sharing behavior and online consultation-offering behavior of physicians, and the review-posting behavior and satisfaction-rating behavior of patients [4,5]. Patients can find a suitable physician for consultation by selecting the type of disease, hospital or department respectively in OHCs. Due to the distance and trust in the local hospital, the patient will first select the hospital and then the department. In this study, we abbreviate the focal physician as the F-physician, and the focal physician’s colleagues who work in the same hospital and department as F-peers. In OHCs, the details of physicians in the same department, including professional title, the number of online consultations and the number of scientific articles published, are listed together. This layout offers physicians a channel to maintain both relationships and competition with their peers from offline to online, and also offers patients an opportunity to make a comparison between physicians. Existing research has shown that people’s behavior can be largely affected by their peers [6,7]. What is more, in the participation behavior of physicians, the online consultation-offering behavior is passive because it is initiated by patients. The knowledge-sharing behavior is spontaneously shared by physicians, so it is proactive. Therefore, the impact of the knowledge-sharing behavior of F-peers on the participation behavior of the F-physician has more research significance than that of F-peers’ online consultation-offering behavior [8]. Similarly, patients can see all other patients’ reviews and satisfaction with physicians, thereby the number of reviews and satisfaction posted by the focal physician’s patients (F-patients) may be affected by patients of the focal physician’s peers (F-P-patients).

Although OHCs grow rapidly, it is hard to maintain OHCs’ sustainable development, because the participation of physicians and patients in OHCs are insufficient and decreasing. Most of research on users’ participation behavior focuses on physicians or patients themselves from intrinsic motivation and extrinsic motivation perspectives, while little research has investigated influencing factors of the F-physician and F-patients’ participation behavior from the perspective of F-peers and F-P-patients. In addition, it is not clear how the knowledge-sharing intensity of F-peers and the department reputation moderate the relationship between peers’ participation behavior and the participation behavior of the F-physician and F-patients. Aiming to fill this research gap, this study focuses on the peer effect of physicians in the same department and the peer effect of patients, by analyzing the impact of F-peers on the F-physician, and F-P-patients on F-patients. At the same time, this study explores the moderating effects of the intensity of F-peers’ knowledge-sharing behavior and the department reputation on the relationship between the participation behavior of F-peers and the F-physician, as well as F-P-patients and F-patients.

For clarity, we use the F-physician for the focal physician, F-peers for colleagues of the focal physician, F-patients for patients of the focal physician, F-P-patients for patients of the focal physician’s colleagues. Participation behaviors of physicians in this study include online consultation offering and knowledge sharing behaviors, while participation behaviors of patients include review posting and satisfaction rating behaviors. Formally, we explore the following research questions:

RQ1: How does the participation behavior of F-peers affect the F-physician’s participation behavior?
RQ2: How does the participation behavior of F-P-patients affect F-patients’ participation behavior?
RQ3: How does the intensity of F-peers’ knowledge sharing behavior and the department reputation moderate the relationship between peers’ participation behavior and the participation behavior of the F-physician and F-patients?

**Figure 1:**
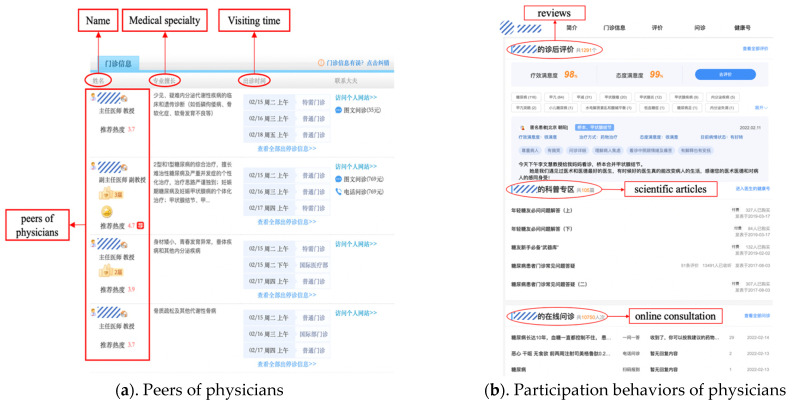
The partial layout and functions of haodf.com, accessed on 1 January 2019.

The partial layout and functions of haodf.com, accessed on 1 January 2019 is shown in Figure 1.

### 1.2. Related Work

#### 1.2.1. Physician Participation Behavior

The participation behavior of physicians in OHCs mainly includes online consultation offering and knowledge sharing. For physicians’ online consultation offering behavior, the extant research mainly focuses on intrinsic motivation and extrinsic motivation [9]. Some literature mainly focuses on the impact of personal intrinsic motivation, such as reputation [10,11,12] and social image [13,14] on physicians’ online consultation services offering. As an extrinsic motivation [15,16], financial incentives can promote physicians to provide free consultation services [17], but it has a negative effect on physicians who share knowledge more actively [18]. For physicians’ knowledge sharing behavior, the existing literature has not only investigated its impact on reputation and performance [19,20,21], but also studied its motivation from different aspects, such as material incentives [22], reputation [23] and so on. Zhang et al. discussed the impact of material motivation and professional motivation on physicians’ information sharing, and found that both material motivation and professional motivation will promote physicians’ information sharing. When physicians’ professional level is high, the role of material incentives will be weakened. Professional knowledge will weaken the impact of material motivation and enhance the impact of professional motivation [24].

#### 1.2.2. Physician Participation Behavior

There are two main processes for patients in OHC: selecting physicians for consultation and evaluating services quality after consultation. This study focuses on the latter, which are the review-posting behavior and the satisfaction-rating behavior of patients [25]. For the motivation of patients’ evaluating-services behavior, on the one hand, previous studies mainly analyze the impact of patients’ perceived services value, which is the subjective evaluation of the utility of consultation services. Online evaluation is an increasingly important source of information for making decisions [26]. More and more patients share diagnosis and treatment experience online or rate the quality of services on the physician rating website [27]. If the expected and actual quality of physicians’ services are found to be inconsistent after receiving the services, patients’ will not be satisfied. On the other hand, existing research finds that the reputation of the hospital can positively affect the relationship between the physician’s reputation and the patient’s posting reviews behavior, while the reputation of F-peers will negatively moderate the relationship between the reputation of the F-physician and the probability of F-patients sharing treatment experience [4].

#### 1.2.3. Peer Effect

Peer effect refers the direct or indirect influence on people of peers, members of social groups with similar conditions [28]. Specifically, it is the impact of peers on focal individual behavior and performance [29,30]. Different peer individuals have heterogeneous peer effects [31,32]. Scholars have studied the impact of peers on the behavior of focal individuals from some perspectives, such as similarity, effort and reputation. Wu et al. found that the reputation of physicians and their peers has a significant impact on the probability of patients sharing treatment experience, and there is a cooperative relationship between physicians and their peers [4].

Extant studies have conducted rich research on physicians’ and patients’ participation behavior in OHCs, but there are still several issues that stay unclear. First of all, most studies focus on the internal and external incentives of physicians’ or patients’ participation behavior in OHCs, but little research investigates the impact of peers’ participation behavior on physicians’ or patients’ participation behavior. Secondly, there is no research that regards physician as an intermediate link and pays attention to the impact of the patients of the focal physician’s peers. Thirdly, most studies have an individual view, instead of an organizational view.

### 1.3. Research Model and Hypotheses

#### 1.3.1. Main Effect Analysis

Based on the accessible–diagnosable and brand extension theories, we explore the impact of F-peers and F-P-patients participation behavior on the F-physician and F-patients participation behavior, and elaborate on a set of research hypotheses.

Accessible–diagnosable theory was put forward by Lynch et al. [33], which integrates the relevant contents of social cognition, cognitive psychology and behavioral decision-making theory. It shows that when information is accessible and can be used for the measure of other products, information spillover will occur [34]. Based on the accessible–diagnosable theory, when physicians share knowledge actively in OHCs, patients will enhance their trust in the physicians, and even the whole department to which the physicians belong. It may bring more potential patients to the department, thereby leading to promote the online consultation-offering behavior of other physicians.

Apart from impacting online consultation services behavior, the knowledge-sharing behavior of physicians might also impact the knowledge-sharing behavior of other physicians. In the same department, physicians not only compare their own abilities and performance with their peers [35], but also tend to imitate the behavior of their peers [36]. When some physicians share knowledge more actively, other physicians will imitate this behavior and share knowledge more actively to narrow the gap with their peers. For ease of reading, we express the focal physician as the F-physician, and the focal physician’s peers as F-peers. Formally, we propose the following hypotheses:

**Hypothesis** **1a.** *The knowledge-sharing behavior of F-peers has a positive impact on the online consultation-offering behavior of the F-physician.*

**Hypothesis** **1b.** *The knowledge-sharing behavior of F-peers has a positive impact on the knowledge-sharing behavior of the F-physician.*

Brand extension theory refers to the idea that a company should market products with good images and use the same brand name in different product categories. When consumers choose a product without knowing its quality, they will refer to the quality of other products under the same brand, and will be affected by positive or negative reviews of other products [37]. This is especially true for experiential products, since consumers cannot perceive the true quality of the products before consumption, such as hotels, restaurants, music products, etc., so brand extension is more important [38]. In OHCs, patients may evaluate their services perceived after the consultation, including sharing their experience, rating the satisfaction level of treatment effect and services attitude, what may provide reference for other patients. Considering that F-peers and the F-physician are colleagues in the same department, the services-evaluating behavior of F-P-patients on F-peers will inevitably affect the services-evaluating behavior of F-patients based on the brand extension theory. Thus, we explore the influence of services-evaluating behavior of F-P-patients on F-patients. The number of reviews and satisfaction by F-P-patients can indicate the reputation of F-peers. Therefore, when patients perceive high reputation of the department, they may display more review-posting behavior and satisfaction-rating behavior [4]. For ease of reading, we express the focal physician’s patients as F-patients, and patients of physician’s peers as F-P-patients. Therefore, we pose two hypotheses as follows:

**Hypothesis** **2a.** *The services-evaluating behavior of F-P-patients has a positive impact on the review-posting behavior of F-patients.*

**Hypothesis** **2b.** *The services-evaluating behavior of F-P-patients has a positive impact on the satisfaction-rating behavior of F-patients.*

#### 1.3.2. Moderating Effect Analysis

The first moderating variable is the intensity of F-peers knowledge-sharing behavior. Studies have demonstrated that the influence of peer ability on focal individual ability is a complex nonlinear relationship [39]. In OHCs, physicians mainly publish scientific articles to share knowledge. The intensity of scientific articles published may vary with physicians, which may reflect the professional ability of physicians in some way. This mechanism can be described in the signal theory. Signal theory includes two important parts, i.e., signaling model and screening model, among which the signaling model refers to one party conveying some information about itself to the other party [40]. In our context, physicians may convey their professional abilities through the behavior of knowledge sharing [22,24]. The higher the intensity of physicians’ knowledge sharing, the more professional capabilities of physicians that patients may percept, thereby the more potential patients may be attracted. This will positively affect the online consultation offering of the F-physician. On the other hand, when the physician feels the more active efforts of his peers, he may imitate them and enhance his knowledge-sharing behavior.

**Hypothesis** **3.** *The knowledge-sharing intensity of F-peers will enhance the influence of knowledge-sharing behavior on the F-physician’s participation behavior (e.g., online consultation offering and knowledge sharing).*

In addition, the knowledge-sharing intensity of F-peers might affect the relationship between the participation behavior of F-patients and F-P-patients. The screening model refers to that when the information of the two parties is asymmetric, one party with less information tries to further insight into the private information of the other party to reduce the information asymmetry [41]. In our context, F-patients consider F-peers’ knowledge-sharing behavior to measure the reliability of F-P-patients evaluation of F-peers. Patients can judge the ability of physicians according to scientific articles published by physicians. Simultaneously, the review-posting behavior and satisfaction-rating behavior of F-P-patients can reflect the online reputation of F-peers [42]. They convey the ability information of physicians from different angles to patients. When F-peers’ knowledge-sharing intensity is consistent with F-P-patients’ services-evaluating behavior, the trust of patients in the overall department is stronger [43], which will positively affect F-patients’ review-posting behavior and satisfaction-rating behavior. Formally, we propose the following hypothesis:

**Hypothesis** **4.** *The knowledge-sharing intensity of F-peers will positively moderate the relationship between the participation behavior of F-P-patients and the participation behavior of F-patients (e.g., review posting and satisfaction rating).*

The second moderating variable is the department reputation. Generally, in the brand extension theory, the well-known product is called the “parent-brand”, and other products of the same organization are called “sub-brand”. In OHCs, the physician’s department is the “parent-brand”, and the physician’s personal services is the “sub-brand”. Based on the brand extension theory, consumers’ trust and recognition of the parent-brand will be extended to the sub-brand [44,45]. Thus, the better reputation of the whole department, the more patients will trust the physicians in the department. This extension may bring potential patients to the department and promote the increase of physicians’ consultation volume. On the other hand, physicians may have a higher sense of collective responsibility in a department with a higher reputation, so they will invest more time and energy in OHCs. When peers have more knowledge-sharing behaviors, physicians’ collective responsibility may prompt them to produce more knowledge-sharing behaviors.

**Hypothesis** **5.** *The department reputation will enhance the influence of F-peers’ knowledge-sharing behavior on the F-physician’s participation behavior (e.g., online consultation offering and knowledge sharing).*

Furthermore, Wu et al. concluded different hospital levels have different degrees of impact on patients’ review posting behavior [4]. Similarly, because the peer effect studied in this paper is based on the same department, the department reputation will affect the trust and satisfaction of the physician’s online consultation services by patients [46]. When patients perceive the high quality of overall physicians in the department, they are more likely to post services experience and receive satisfactory services, thereby enhancing the positive influence of F-P-patients’ services-evaluating behavior on the review-posting behavior and the satisfaction rating behavior of F-patients. Thus, we put forward the following hypothesis:

**Hypothesis** **6.** *The department reputation will positively moderate the relationship between the participation behavior of F-P-patients and the participation behavior of F-patients (e.g., review posting and satisfaction rating).*

**Figure 2:**
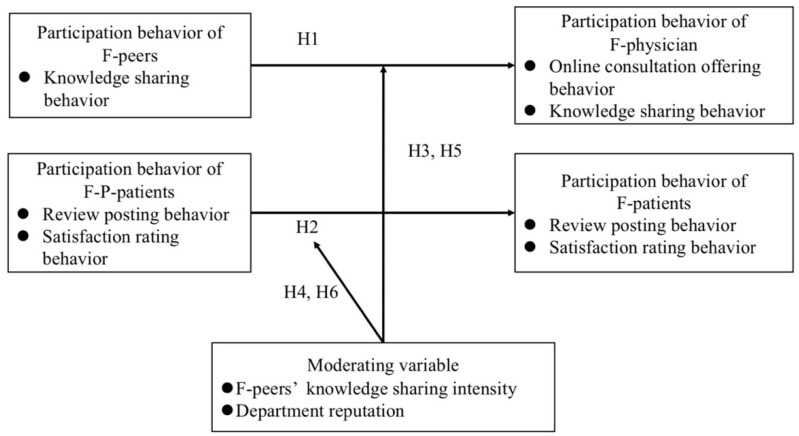
Conceptual model.

In this study, the hypotheses are established according to the relationships expressed in Figure 2. From the perspective of the physician’s peers in the same hospital and department, this study discusses the impact of F-peers’ participation behavior on the online consultation-offering behavior and knowledge-sharing behavior of the F-physician, and the impact of F-P-patients’ participation behavior on the review-posting behavior and the satisfaction-rating behavior of F-patients. In addition, we also deeply study the knowledge sharing intensity of F-peers and the department reputation on physicians’ and patients’ participation behavior. By studying peers of physicians and patients, OHCs can consider the influence of peer effect when establishing the incentive mechanism for physicians’ and patients’ participation behavior. Our findings may contribute to the sustained, efficient and stable development of OHCs.

## 2. Materials and Methods

### 2.1. Research Design

We chose Haodf.com as the research context. Haodf.com, established in 2006, is one of the pioneering OHCs in China, which has functions such as online and offline consultation, telemedicine, appointment registration, disease management and popularization of medical knowledge. To a certain extent, it alleviates the problem of difficult and expensive medical treatment. By April 2021, Haodf.com has included 794,235 physicians from 9656 hospitals, and the scale of users is still expanding. It has gradually become an important platform to help patients solve disease problems online.

Compared with traditional offline medical care, OHCs are especially beneficial for patients who need long-term management of chronic diseases. Patients can communicate with physicians at home at any time about their physical conditions, medication status, disease progress and treatment plans. In addition, the outbreak of the COVID-2019 has further promoted the development and growth of OHCs. Due to the needs of the epidemic, patients can consult and communicate with physicians online and obtain prescription drugs through OHCs without entering actual hospitals. It can not only effectively reduce the pressure on hospitals, but also reduce the contact between physicians and patients, and try to avoid the threat of cross infection.

Considering the uniqueness of each physician, there may be missing characteristics that do not change with time but change with individuals, such as the personalities, hospital level, physician title, etc. Moreover, there may also be missing characteristics that do not change with individuals but change with time. For example, the results of a certain month may be affected by the impact of external events. The value *p* in Hausman test was 0.0000, indicating that the original hypothesis was strongly rejected. Thus, we use the two-way fixed effect model to estimate and control the individual heterogeneity effect and time heterogeneity effect. In addition, we also conducted a robust test to verify the stability of the results by negative binomial regression model.

### 2.2. Data and Variables

Considering that patients mainly consult regarding conventional diseases on the OHCs and sufficient data, we selected two departments of Neurology and Gastroenterology, which are the most consulted by patients, and used Python to trawl through the 12-month data of the Haodf.com platform from January 2019 to December 2019. We collected data on the participation behavior of physicians and their colleagues in the department, including personal information, the number of online consultations, the number of scientific articles published, the number of reviews and patient satisfaction with the services. After obtaining the original data, we cleaned the data. First, based on the time span in this paper, we selected the physicians registered before January 2019, and deleted the data of physicians who have no colleagues or have not established a personal homepage during our study period. Then, each physician’s data and his peers’ data were matched according to whether they are in the same hospital and department. Finally, 39,564 valid physicians’ data were obtained.

On the Haodf.com platform, physicians and patients are two main types of users. For physicians, they can communicate with patients through online consultation to understand patients’ condition and make treatment plans. In addition, physicians can share knowledge on the platform according to their own experience, for instance, publish scientific articles to show their skills to patients. For patients, they can select a suitable physician for consultation by viewing the information about physicians, including the physician’s professional title, recommendation popularity, number of published articles, reviews and online consultations. After receiving the consultation services, patients may post reviews to share their experiences, and rate their satisfaction with physicians.

The definitions and descriptive statistics of the key variables are listed in Table 1. Dependent variables are the F-physician’s and F-patients’ participation behavior. Specifically, the participation behavior of physicians refers to knowledge sharing (*Art_Nm_it_*) and online consultation services offering (*Consult_it_*), while the participation behavior of patients refers to review posting and satisfaction rating, which are characterized by the number of reviews (*Com_All_it_*) and satisfaction rating (*S_All_it_*), respectively. Our independent variables are F-peers’ and F-P-patients’ participation behavior. The participation behavior of F-peers is knowledge sharing (*Pr_Art*_*it−*1_). Due to the fact that physicians may have different personal preferences when publishing articles in the OHC, some physicians prefer to integrate relevant health knowledge into one article and publish it, while some physicians opt to publish it separately. Therefore, in order to avoid this error, variable *Pr_Art*_*it−*1_ is measured by whether peers of the physician i published an article in month t. The participation behavior of F-P-patients is reviews *posting*, which is characterized by the number of reviews (*P_All*_*it−*1_) and satisfaction rating (*P_Q*_*it−*1_). Considering that some physicians have a large number of services reviews, while some have a small number of reviews, we use the average number of services reviews of peers to measure the variable *P_All*_*it−*1_. In addition, we control some physicians’ individual factors, such as the number of physicians’ offline services reviews, the satisfaction of physicians’ offline services, the number of physicians’ online services reviews, and the satisfaction of physicians’ online services. The number of scientific articles published of the F-physician’s is a control variable when discussing the impact of F-peers’ participation behavior on the number of the F-physician’s online consultation offering. The offline services satisfaction in the Haodf.com platform is mainly composed of two parts: curative effect satisfaction and attitude satisfaction. The average of curative effect satisfaction and attitude satisfaction is taken as offline services satisfaction. The reviews of patients can be portrayed by the number of reviews and satisfaction with medical services.

### 2.3. Empirical Model

We explore the impact of F-peers’ knowledge-sharing behavior on the F-physician’s participation behavior. The empirical models are shown as follows:

(1) $$\begin{array}{ll} lnConsult_{it}= & \beta_{1}Pr\_Art_{it-1}+\beta_{2}Comt_{it-1}+\beta_{3}Cmt\_Q_{it-1}+\beta_{4}Vote_{it-1}+\beta_{5}Vote\_Q_{it-1}+\beta_{6}Self\_Art_{it-1}\\ & +\lambda_{i}+\gamma_{i}+\mu_{it} \end{array}$$

(2) $$Art\_Nm_{it}=\alpha_{1}Pr\_Art_{it-1}+\alpha_{2}Comt_{it-1}+\alpha_{3}Cmt\_Q_{it-1}+\alpha_{4}Vote_{it-1}+\alpha_{5}Vote\_Q_{it-1}+\lambda_{i}+\gamma_{i}+\mu_{it}$$

The dependent variable in Equation (1) is the logarithm of the amount of the F-physician’s online consultation services in *t* month due to the standard deviation and value range, while the dependent variable in Equation (2) is the total number of scientific articles published by the F-physician in *t* month [7]. The independent variables in both Equations (1) and (2) are whether F-peers publish articles, and others are the control variables. The meaning of all variables is shown in Section 2.2.

We analyze the impact of the participation behavior of F-P-patients on the review-posting and satisfaction-rating behavior of F-patients. We use two variables to measure patients’ behavior, the number of reviews and satisfaction ratings [4]. The estimated equation is defined as follows:

(3) $$Result_{it}=\beta_{1}P\_All_{it-1}+\beta_{2}P\_Q_{it-1}+\beta_{3}Consult_{it-1}+\beta_{4}Art\_Nm_{it-1}+\lambda_{i}+\gamma_{i}+\mu_{it}$$

where *Result_it_* represents the outcome variables *Com_All_it_* and *S_All_it_*, which are the total number of reviews and satisfaction by F-patients, respectively. The independent variable of Equation (3) is the average number of reviews and satisfaction ratings by F-P-patients in the *t−1* month. In Equations (1)–(3), *λ_i_* is the intercept term of individual fixed effect. *γ_i_* represents the intercept term of time fixed effect, which is described by the time dummy variable. *β_(_*_1*−*6)_ and *α_(_*_1*−*5)_ are the regression coefficient. *μ_it_* represents the random disturbance term.

## 3. Results

### 3.1. Effect on the F-Physician and F-Patients

In this study, we mainly explore the impact of F-peers’ knowledge sharing and the services evaluating of F-P-patients on the participation behavior of the F-physician and F-patients. The estimation results of Hypotheses 1 and 2 are shown in Table 2 and Table 3, respectively, in which M1 only contains control variables, and M2 adds independent variables on the basis of M1.

From the M2 results of Hypothesis 1a in Table 2, it can be seen that the coefficient of the variable Pr_Artit−1 is positive and significant, with a coefficient of 0.039. It shows that when F-peers publish scientific articles, the F-physician’s consultation is 3.9% more than that when F-peers do not publish scientific articles. Therefore, Hypothesis 1a is supported. The M2 of Hypothesis 1b in Table 2 shows that the coefficient of the variable Pr_Artit−1 is positive and significant, with a coefficient of 0.12. It suggests that when F-peers published scientific articles, the number of articles published by the F-physician is an average of 0.12 more. Hypothesis 1b is supported.

From the results in Table 3, the services evaluating behavior (the number of reviews and satisfaction) of F-P-patients has a positive effect on F-patients’ reviews posting (*β*_1_ = 0.114, *β*_2_ = 0.159) and F-patients’ satisfaction (*α*_1_ = 0.019, *α*_2_ = 0.190). According to the results in the column of variable *Com_All_it_*, it can be concluded that when F-P-patients send 1 review, F-patients may send 0.114 reviews. At the same time, when the satisfaction of F-P-patients increases by 1 point, F-patients will send 0.159 reviews. Similarly, according to the results in the column of variable *S_All_it_*, the satisfaction of the F-physician will be increased by 0.019 and 0.19 respectively when F-P-patients has one more review or the satisfaction is increased by 1 point. Therefore, both Hypotheses 2a,b hold true.

### 3.2. Moderation Effect on the F-Physician and F-Patients

Besides the main effects, we also study the moderating effect of the intensity of F-peers’ knowledge sharing and the department reputation. The estimation results of Hypothesis 3–6 are omitted. For Hypothesis 3, the coefficient of the interaction term *Pr_Art_it−_*_1_ *× P_AtNm_it−_*_1_ is significant and positive (*β*_2_ = 0.052). In contrast, as shown in column *Art_Nm_it_* of Table 4, the coefficient of the interaction term *Pr_Art_it−_*_1_ *× P_AtNm_it−_*_1_ is significant but negative (*α*_2_ = −0.010). It indicates that the intensity of F-peers’ knowledge sharing positively moderate the impact of F-peers’ knowledge sharing on the F-physician’s online consultation, whereas they negatively moderate that on the F-physician’s knowledge sharing. Therefore, Hypothesis 3 is partially supported.

For Hypothesis 4, according to the estimated result that the explained variable is *Com_All_it_*, only the interaction coefficient *P_All_it−_*_1_ *× P_AtNm_it−_*_1_ is significantly positive. For the estimated result of the variable *S_All_it_*, the interaction coefficients of the intensity of F-peers’ knowledge sharing are not significant. It indicates that only F-P-patients’ review posting has a bigger effect on F-patients’ review posting when the intensity of F-peers’ knowledge sharing is higher. Therefore, Hypothesis 4 is partially supported.

For Hypothesis 5, the coefficient of the interaction term *Pr_Art_it−_*_1_ *× P_Nhd_it−_*_1_ are negative. It demonstrates that the higher the department reputation, the more it will weaken the effect of F-peers’ knowledge sharing on the F-physician’s online consultation and knowledge sharing. Thus, Hypothesis 5 is not supported.

For Hypothesis 6, the moderation effects are significant at the level of 0.01 (*β*_3_ = 0.056, *β*_4_ = 0.093). According to the estimation results in the column *S_All_it_*, the interaction item *P_Q_it−_*_1_* P_Nhd_it−__1_ is positive and significant, while the other interaction coefficient is not significant. It demonstrates that when the department reputation is higher, the effect of F-P-patients’ satisfaction rating on the participation behavior of F-patients is bigger, but the effect of F-P-patients’ review posting only has a bigger effect on the satisfaction rating of F-patients. Therefore, Hypothesis 6 is partially supported.

### 3.3. Robust Test

In order to verify the stability of the results, we conducted robust test. Considering that the dependent variables in Hypothesis 1a, b are both non-negative integers, which are the number of the F-physician’s consultations and the number of published articles, respectively, the estimations can be counted by models such as Poisson regression and negative binomial regression. Table 4 gives a detailed description of the two dependent variables. The mean of both is less than the variance, which is more suitable for the negative binomial regression model. Thus, we chose the panel negative binomial regression model for our robust test.

According to the robust results of Hypothesis 1, the coefficient of the variable *Pr_Art_it−_*_1_ is positive, and the F-physician’s consultation volume increased by an average of 0.044 after F-peers published scientific articles. Similarly, the coefficient of the variable *Pr_Art_it−_*_1_ also is significant and positive. The number of articles published by the F-physician after F-peers published scientific articles is an average of 0.23 more. Hence, they are consistent with the results in Table 2, which proves that the results are robust.

Next, we test the robustness of Hypothesis 2. We refined the services-evaluating behavior of patients into online services-evaluating behavior and offline services-evaluating behavior, and studied their impact on the number of reviews and satisfaction of F-patients. P_Cmt_it−1_ is defined as the number of online services reviews by F-P-patients, and P_Cq_it−1_ is the satisfaction of online services. In offline services evaluation, P_Vt_it−1_ represents the number of reviews of F-P-patients, and P_Vq_it−1_ is their satisfaction. According to the robust results of Hypothesis 2, the services evaluating of F-P-patients, both online and offline, has a positive impact on the number of reviews and satisfaction by F-patients, which is consistent with the results in Table 3, proving that the results are robust.

## 4. Discussion

This study mainly investigates the effect of F-peers’ behavior on the F-physician’s behavior, as well as F-P-patients’ behavior on F-patients’ behavior in OHCs. On the basis of the accessible–diagnosable and brand extension theories, we developed six research hypotheses and established empirical models accordingly. This work obtains four key findings. (1) We found that the knowledge-sharing behavior of F-peers has a positive effect on both the online consultation-offering and knowledge-sharing behavior of the F-physician. (2) The services evaluating of F-P-patients has positive effects on both the review posting and satisfaction rating of F-patients. (3) The intensity of F-peers’ knowledge sharing positively moderate the effect of F-peers’ knowledge sharing on the F-physicians’ online consultation offering; However, it has no moderating effect on the relationship between knowledge sharing of F-peers and the F-physician. (4) The department reputation negatively moderates the impact of F-peers’ knowledge sharing on the F-physician’s participation, whereas it has a positive effect on the relationship of services evaluating of F-P-patients and F-patients, Nevertheless, it has no effect on the relationship between F-P-patients’ services evaluating behavior and F-patients’ satisfaction.

In this paper, we study the participation behavior of physicians and patients from the perspective of peer effect from colleagues and other patients. Some existing research conclude that when competitive enterprises nearby actively interact with consumers, the target enterprises may be negatively affected in performance, but may also benefit from the attracted passenger flow [47,48]. Through our empirical analysis, we find that the physician can also benefit from his/her colleagues, although they are competitors when patients choose physicians for consultation. This may because when focal physician’s patients perceive the more positive attitude of focal physician’s colleagues, they may trust the focal physician more, causing the online consultation volume of the focal physician further improved. This may help the communication between patients and physicians in a potential way.

Moreover, some of the colleagues’ behaviors will moderate the relationship between the behaviors of the colleagues and the focal physician. This phenomenon indicates that the active behaviors of colleagues will send a strong quality signal to patients that the department’s overall ability is great. Additionally, some of the colleagues’ behaviors also moderate the relationship between the behaviors of colleagues’ patients and the focal physician’s patients. When the physician’s patients perceive the overall quality and reputation of the department from multisource quality signals, they are more inclined to be active in OHCs.

Previous studies have found the effectiveness of physicians’ information, services quality and patients’ expectations of consultation will affect the number and satisfaction of patients’ evaluations [49,50,51]. In this paper, we find that the participation behavior of colleagues’ patients has a positive effect on the participation behavior of the focal physician’s patients. According to the accessible–diagnosable theory, when there is a large volume of good feedback by colleagues’ patients, the physician’s patients will think that physicians in the same department may have a higher reputation, thereby increasing their trust in the focal physician and further promoting their positive experience with the focal physician. The result also confirms the internal influence between patients.

Furthermore, as the “parent-brand” in OHCs, patients’ recognition of the department should improve their trust in physicians [21]. However, we find that the department reputation will negatively moderate the impact of colleagues’ knowledge-sharing behavior on the physician’s participation behavior. A possible reason is that there may be physicians who feel that it is unfair that they have not received the annual “Good Physician” medal even with hard work since the competition is severe in a high-reputation department, which reduces their motivation. In contrast, we also find that the department reputation may enhance the impact of the behavior of colleagues’ patients on the behavior of the Focal physician’s patients. This may because the patient’s attitude towards the parent brand, the department, will be transferred to the sub-brand, physicians, of the same department, according to the brand extension theory.

## 5. Conclusions

### 5.1. Research Contributions

The contributions of this study are threefold. Firstly, we investigated the peer effect of from the perspective of the physician’s colleagues and colleagues’ patients in OHCs. Most studies focus on the internal and external incentives of physicians’ or patients’ participation behavior in OHCs, but little research investigates the impact of peers’ participation behavior on physician’s or patients’ participation behavior. This study enriches the existing literature on the peer effect, and refines the peer effect into participation behaviors of physicians (online consultation offering and knowledge sharing) and patients (review posting and satisfaction rating) in OHCs.

In addition, we construct the conceptual framework of different types of individual participation behaviors in OHCs, and explore the interaction between multiple objectives’ participation behaviors. There is no research that regards the physician as an intermediate link and pays attention to the impact of the patients of the focal physician’s colleagues. Our study explores the peer effect of physicians and patients at the same time, which expands the one-sidedness of existing studies that only focus on a single individual participation behavior.

Finally, the previous literature pays less attention to the impact of the department reputation on the participation behavior of other individuals. Actually, physicians do not exist alone in OHCs, and their organization may also have an impact on physicians and patients. Therefore, this study focuses on the impact of department reputation, and analyzes its regulatory effect on users’ participation behavior from a higher organizational level.

### 5.2. Practical Implications

This paper studies the influence of F-peers and F-P-patients’ participation behavior on the participation behavior of the F-physician and F-patients, and constructs an influence mechanism of users’ participation behavior, which has certain practical significance for physicians, patients and platforms. Therefore, on the basis of the empirical results, this paper puts forward relevant suggestions from the following perspectives.

For platform of OHCs, it is necessary to pay more attention to the competitive and cooperative relationship between physicians and their colleagues. Since active physicians in OHCs will influence the participation behavior of other physicians, incentives and reminders should be introduced to physicians. On the other hand, the platform should pay more attention to services reviews posted by patients. The number of reviews by colleagues’ patients will positively affect the focal physician’s patients’ review posting behavior, so the platform can develop incentive mechanisms to encourage patients to post more reviews. For example, the platform can send reminder messages to patients after the consultation service ends, and reward patients who complete a review.

For physicians, they can show their professional ability to patients by actively publishing scientific articles. Improving the quality of colleagues will also bring potential patients for physicians themselves, so physicians can choose the department with a high number of senior physicians when applying for a job. In addition, physicians should actively reply to patients’ reviews and give them treatment suggestions, to build up reputation. Considering that patients’ evaluations may attract other potential patients to ask for consultation, physicians should promptly remind patients to post their reviews after both online and offline consultation. At the same time, when replying to patients, they also should pay attention to their tone and attitude to improve patients’ satisfaction with their services.

For patients, when choosing a physician, they can acquire information about the number of published scientific articles and other patients’ reviews of the physician, and try to choose a physician with high overall departmental quality for consultation. On the other hand, patients should also take the initiative to share their experiences after the consultation services, so as to provide reference for other patients and promote the sustainable development of OHCs.

### 5.3. Limitations and Future Research

This study has the following deficiencies, which provide new prospects for future research. (1) This paper selected physicians of two types of diseases: neurology and gastroenterology. It is limited as we cannot verify whether the research conclusion can be extended to physicians/patients of other disease types. In future research, we can select more disease types for analysis, classify disease types and study the participation behavior of physicians and patients under different disease types. (2) This study selected monthly panel data for a full year in 2019 for model estimation and analysis. However, due to the impact of the COVID-19, physicians and patients’ engagement in OHCs may be increase. Therefore, future research can expand the time period and study the users’ participation behavior in the context of the COVID-19. (3) This paper uses panel negative binomial regression model for robustness testing to deal with endogeneity problems. However, there are still some deficiencies in causal inference. Future research can conduct more abundant research through methods such as randomized experiments, and more accurately estimate the peer effect of physicians’ and patients’ participation behavior in OHCs. (4) Although this study focused on the number of evaluations and satisfaction of services, the patients’ services evaluations are diverse, which can be deeply analyzed from multiple angles. Therefore, future research can focus on the emotional tendency and readability of services evaluations, etc.

## Figures and Tables

**Table 1:** Descriptive of variables.

Variable	Definition	Mean	SD	Min	Max
**Dependent Variable**					
*Consult_it_*	Number of online consultations for a physician *i* in month *t*.	8.163	32.851	0	4236
*Art_Nm_it_*	Number of scientific articles published for a physician *i* in month *t*.	0.183	2.307	0	217
*Com_All_it_*	Total number of reviews for a physician *i* in month *t*.	1.21	4.681	0	135
*S_All_it_*	Average satisfaction of reviews for a physician *i* in month *t*.	1.353	1.667	0	4
**Independent Variable**					
*Pr_Art*_*it−*1_	Binary variable equals to 1 if peer of a physician *i* published articles in month *t−*1; otherwise it equals 0.	0.245	0.43	0	1
*P_All*_*it−*1_	Average number of services reviews for peer of a physician *i* in month *t*−1.	0.733	1.728	0	78
*P_Q*_*it−*1_	Average satisfaction of services reviews for peer of a physician *i* in month *t−*1.	0.796	0.787	0	4
**Control Variable**					
*Self_Art*_*it−*1_	Binary variable equals to 1 if a physician *i* published articles in month *t−*1; otherwise it equals 0.	0.047	0.211	0	1
*Comt*_*it−*1_	Total number of online services reviews for a physician *i* in month *t−*1.	16.515	70.583	0	1543
*Cmt_Q*_*it−*1_	Average satisfaction of online services reviews for a physician *i* in month *t−*1.	0.376	0.478	0	1
*Vote*_*it−*1_	Total number of offline services reviews for a physician *i* in month *t−*1.	28.851	77.357	0	1400
*Vote_Q*_*it−*1_	Average satisfaction of offline services evaluations for a physician *i* in month *t−*1.	0.976	1.386	0	3

**Table 2:** Two-way fixed effect of Hypothesis 1.

	Hypothesis 1a		Hypothesis 1b	
Variable	M1	M2	M1	M2
*Self_Art*_*it−*1_	0.363 ***	0.361 ***		
	(22.56)	(22.46)		
	*p* < 0.001	*p* < 0.001		
*Comt*_*it−*1_	−0.002 ***	−0.002 ***	−0.006 ***	−0.006 ***
	(−3.66)	(−3.62)	(−3.77)	(−3.73)
	*p* < 0.001	*p* < 0.001	*p* < 0.001	*p* < 0.001
*Cmt_Q*_*it−*1_	0.058 ***	0.058 ***	0.073	0.072
	(4.42)	(4.40)	(1.58)	(1.56)
	*p* < 0.001	*p* < 0.001	*p* = 0.114	*p* = 0.119
*Vote*_*it−*1_	0.001 ***	0.002 ***	0.004 **	0.004 **
	(2.63)	(2.67)	(2.04)	(2.07)
	*p* = 0.008	*p* = 0.007	*p* = 0.041	*p* = 0.038
*Vote_Q*_*it−*1_	0.041 ***	0.041 ***	0.007	0.008
	(8.80)	(8.88)	(0.44)	(0.51)
	*p* < 0.001	*p* < 0.001	*p* = 0.660	*p* = 0.610
*Pr_Art*_*it−*1_		0.039 ***		0.120 ***
		(4.26)		(3.74)
		*p* < 0.001		*p* < 0.001
Constant	0.943 ***	0.932 ***	0.134 ***	0.101 ***
	(68.63)	(66.77)	(2.77)	(2.05)
	*p* < 0.001	*p* < 0.001	*p* = 0.006	*p* = 0.040
Observations	39,564	39,564	39,564	39,564
R-squared	0.824	0.824	0.328	0.328
Number of ID	3297	3297	3297	3297

t-statistics in parentheses, *** *p* < 0.01, ** *p* < 0.05, * *p* < 0.1.

**Table 3:** Two-way fixed effect of Hypothesis 2.

	Hypothesis 2a		Hypothesis 2b	
Variable	M1	M2	M1	M2
*Art_Nm*_*it−*1_	0.018 ***	0.018 ***	0.002	0.002
	(3.48)	(3.44)	(1.16)	(1.19)
	*p* < 0.001	*p* < 0.001	*p* = 0.246	*p* = 0.234
*Consult*_*it−*1_	0.014 ***	0.014 ***	0.0004 ***	0.0004 **
	(31.21)	(31.09)	(2.66)	(2.46)
	*p* < 0.001	*p* < 0.001	*p* = 0.008	*p* = 0.014
*P_All*_*it−*1_		0.114 ***		0.019 ***
		(6.64)		(3.02)
		*p* < 0.001		*p* = 0.003
*P_Q*_*it−*1_		0.159 ***		0.190 ***
		(3.91)		(13.03)
		*p* < 0.001		*p* < 0.001
Constant	1.046 ***	0.901 ***	0.685 ***	0.594 ***
	(29.95)	(22.38)	(54.65)	(41.18)
	*p* < 0.001	*p* < 0.001	*p* < 0.001	*p* < 0.001
Observations	39,564	39,564	39,564	39,564
R-squared	0.030	0.032	0.236	0.240
Number of ID	3297	3297	3297	3297

t-statistics in parentheses, *** *p* < 0.01, ** *p* < 0.05, * *p* < 0.1.

**Table 4:** Descriptive Statistics.

Variable	Obs	Mean	Std. Dev.	Variance	Skew.	Kurt.
*Art_Nm_it_*	39564	0.183	2.307	5.322	51.919	3867.941
*Consult_it_*	39564	8.163	32.851	1079.208	57.297	6964.579

## Data Availability

The data presented in this study are available on request from the corresponding author.
